# Inhibition of PFKFB Preserves Intestinal Barrier Function in Sepsis by Inhibiting NLRP3/GSDMD

**DOI:** 10.1155/2022/8704016

**Published:** 2022-12-23

**Authors:** Yongsheng Zhang, Yukun Liu, Zhenxing Xie, Qinxin Liu, Yangfan Zhuang, Weiming Xie, Xiang Wang, Wei Gao, Fan Yang, Zhanfei Li, Xiangjun Bai, Yuchang Wang

**Affiliations:** ^1^Trauma Center/Department of Emergency and Traumatic Surgery, Tongji Hospital of Tongji Medical College, Huazhong University of Science and Technology, Wuhan 430030, China; ^2^Department of Plastic and Cosmetic Surgery, Tongji Hospital, Tongji Medical College, Huazhong University of Science and Technology, Wuhan 430030, China

## Abstract

Intestinal barrier dysfunction is associated with the occurrence and development of sepsis. Further, aerobic glycolysis plays an essential role in inflammation and cell death. This study is aimed at investigating the protective effect and mechanism of PFKFB3 inhibition on intestinal barrier dysfunction in sepsis mice. Sepsis mouse models were established by cecal ligation and puncture (CLP) in wild-type mice and Gsdmd^−/−^ mice. The results showed that the expression of 6-phosphofructo-2-kinase/fructose-2,6-biphosphatase 3 (PFKFB3) in the small intestines was significantly upregulated in sepsis. 3-(3-Pyridinyl)-1-(4-pyridinyl)-2-propen-1-one (3PO), the specific inhibitor of PFKFB3, and Gsdmd gene knockout significantly inhibited the inflammatory response and cell death caused by sepsis, thus alleviating intestinal damage and barrier dysfunction. 3PO was also shown to significantly inhibit oxidative stress and NLRP3/caspase-1/GSDMD-dependent cell pyroptosis in the small intestines. The in vitro studies revealed that 3PO reduced NLRP3/caspase-1/GSDMD-dependent cell pyroptosis by inhibiting ROS. Taken together, our results suggest that PFKFB3 is involved in inflammation, oxidative stress, and pyroptosis during sepsis and enhances intestinal damage, which may provide important clues about the potential targets to be exploited in this highly lethal disease.

## 1. Introduction

Sepsis is a life-threatening organ dysfunction caused by a dysregulated host response to an infection and is a major cause of admission and death in intensive care units [1]. The gut is considered to play a crucial role in the pathophysiology of sepsis by functioning as the “motor” of the systemic inflammatory response [2]. Intestinal barrier dysfunction leads to microbial translocation, which plays a vital role in secondary infection and sepsis [3, 4]. Therefore, exploring new effective therapeutics against intestinal injury in sepsis is of great significance.

Aerobic glycolysis is an important metabolic pathway controlled by a variety of glycolytic enzymes. It plays an essential role in tumors and sepsis [5, 6, 7]. Among the PFKFB isoenzymes, PFKFB3 mainly relies on glycolysis to produce ATP, regulating glycolytic flux and fructose-2,6-BP (Fru2,6-BP). Fru2,6-BP is an effective allosteric regulator of the key glycolytic enzyme phosphofructokinase 1 (PFK-1) [8]. PFKFB3 is widely expressed in human tissues and has been shown to affect the physiological and pathological activity of various tissues. PFKFB3 has been shown to protect against diet-induced insulin resistance and inflammation in adipose tissues [9]. PFKFB3 is also involved in the regulation of glycolysis, cell viability, and proliferation in tumor cells. Studies have found that 3PO, the specific inhibitor of PFKFB3, inhibits the production of lactic acid by glycolysis and inhibits tumor growth [10, 11, 12]. In addition, aerobic glycolysis has also been shown to play an essential role in sepsis. 3PO can reduce sepsis-related lung injury, including acute lung injury (ALI), by inhibiting inflammation and epithelial cell apoptosis [7]. Hence, PFKFB3-dependent aerobic glycolysis may act as a key regulator in the pathogenesis of intestinal barrier dysfunction in sepsis.

In the present study, we explored the role and mechanism of PFKFB3 in sepsis barrier dysfunction using PFKFB3 small molecule inhibitor 3PO and Gsdmd^−/−^ mice.

## 2. Materials and Methods

### 2.1. Animals

To systematically compare the protective effects of PFKFB3 inhibition and GSDMD knockout on septic mice, we used GSDMD^−/−^ mice. Gsdmd^−/−^ mice of C57BL/6 genetic background were purchased from Jiangsu Jicui Yaokang Biotechnology Co., Ltd. Six-week-old C57BL/6 wild-type male mice were housed in standard environmental conditions with a 12 h light/12 h dark conditions and fed with standard commercial chow diet with free access to water. The studies involving animals were reviewed and approved by the Animal Ethics Committee of Tongji Hospital, Tongji Medical College, Huazhong University of Science and Technology.

### 2.2. CLP-Induced Sepsis Model

The sepsis model was established by CLP as previously described. Briefly, all experiments used 7-8-week-old male mice (22-26 grams). The mice were anesthetized by an intraperitoneal injection of sodium pentobarbital (40 mg/kg). The sham-operated mice underwent the same procedure without CLP. Finally, the mice were resuscitated by subcutaneous injection of sterile normal saline (50 mL/kg).

### 2.3. Experimental Protocol

C57BL/6J mice were randomly divided into five groups: (1) sham group, (2) CLP group, (3) sham-3PO (50 mg/kg) group, (4) CLP+3PO (50 mg/kg) group, and (5) CLP+Gsdmd^−/−^ group. The dose of 3PO was selected based on a previous experimental study [13]. The mice were euthanized 24 hours after the sham operation or CLP surgery for subsequent experiments. Another group of model animals was selected for treatment with CY-09 (TopScience, USA, T4164) at a dose of 20 mg/kg to reveal the significance of NLRP3 in intestinal injury [14]. LPS (1 *μ*g/mL) was used to induce Caco-2 cell injury in establishing an in vitro model of sepsis. Caco-2 was purchased from the Cell Bank of Shanghai Institutes for Biological Science (Shanghai, China). The intestinal epithelial cell line Caco-2 was cultured in Dulbecco's modified Eagle medium (DMEM) containing 5% fetal bovine serum (FBS), 100 U/mL penicillin, and 100 *μ*g/mL streptomycin at 37°C and 5% CO_2_. Caco-2 cells were incubated with or without 3PO at a dose of 2 mM for 30 min before the model set-up. The Caco-2 cells were incubated with NAC (10 mM) for 30 min before the model set-up to clarify the role of ROS in injury. The in vitro doses of LPS, 3PO, and NAC were selected based on a previous experimental study [7, 15, 16].

### 2.4. Western Blot Analysis

Intestinal tissues were homogenized in a protease inhibitor cocktail containing radioimmunoprecipitation assay (RIPA) lysis buffer (Boster, Wuhan, China). The protein concentration was quantitatively determined using bicinchoninic acid (BCA) protein kit (Boster). The extracted protein was separated by 10% SDS-PAGE and transferred to PVDF membranes. The membranes were blocked with 5% nonfat milk and incubated with primary antibodies (including PFKFB3, NLRP3, caspase-1, GSDMD, IL-18, and IL-1*β*) (1 : 1000, ab, Abcam, USA) overnight at 4°C. After that, the membranes were incubated with rabbit HRP-conjugated secondary antibody at room temperature for 2 h, and the protein bands were visualized using chemiluminescent peroxidase substrate (Millipore, Boston, MA, USA). GAPDH antibody was used as the internal control. Densitometric analysis was quantified using the ImageJ software (National Institutes of Health, USA).

### 2.5. Histological Examination

Intestinal tissues were fixed overnight in 4% paraformaldehyde, embedded in paraffin, and cut into 5 *μ*m thick slices. After that, hematoxylin and eosin (H&E) staining was performed. Histopathological examinations were carried out using a microscope (RX51, Olympus Optical Co., Ltd., Tokyo, Japan).

### 2.6. ELISA

The intestinal tissues were homogenized and centrifuged at 3000 RPM at 4°C for 15 min, and the supernatant was collected. After dilution, the supernatant was placed into the sample wells on the HRP plate. The absorbance was measured at 450 nm using a microplate analyzer (Multiskan Go, Thermo Fisher Scientific, Inc.). Levels of tumor necrosis factor (TNF-*α*), interleukin- (IL-) 6, and IL-1*β* were determined by an ELISA kit (Shenzhen Dawei, China).

### 2.7. Measurement of Intestinal Permeability

Diamine oxidase (DAO) levels were detected by ELISA (YM-S2959, Shanghai Yuanmu Biotechnology Co., Ltd., Shanghai, China). Serum D-lactic acid levels were determined by enzyme coupling spectrophotometry (K667-100, BioVision, California, USA). All experimental procedures were carried out in strict accordance with the laid guidelines.

### 2.8. Immunofluorescence Staining

The sections were stained overnight with rabbit polyclonal antibody against NLRP3 (1 : 200) at 4°C followed by incubation at room temperature for 2 h with a mixture of goat anti-rabbit CY5.5-labeled secondary antibody (Abcam, Cambridge, UK) and DAPI staining for 10 min. Images were then obtained by confocal fluorescence microscopy (RX51, Olympus Optical Co., Ltd., Tokyo, Japan).

### 2.9. Oxidative Stress Assessment

The intestinal tissues were homogenized and centrifuged at 3000 rpm for 10 min, and the supernatant was collected. Reactive oxygen species (ROS), malondialdehyde (MDA), and superoxide dismutase (SOD) were measured using the ROS, MDA, and SOD test kit assay kits (Nanjing Jiancheng Bioengineering Institute) according to the manufacturer's instructions.

### 2.10. Knockdown of GSDMD in Caco-2 Cells

To knock down Gsdmd, Caco-2 cells were transfected with Gsdmd target siRNA or control nonspecific siRNA (Ruibo Biotechnology, China) according to the manufacturer's protocol. The sequences were as follows: si-GSDMD: GCAGGAGCTTCCACTTCTA. After 8 h transfection, LPS (1 *μ*g/mL) stimulated the cells for 6 h and then proceeded with the experiments.

### 2.11. Cell Viability Assay and LDH Detection

Cell viability was assessed using the CCK-8 assay (Beyotime, China) according to the manufacturer's instructions. The absorbance was measured at 450 nm using Thermo Fisher Scientific to evaluate cell viability. LDH was determined an LDH Assay Kit (Beyotime, China) according to the manufacturer's instructions.

### 2.12. Bioinformatic Methodology

To see if the gene is coexpressed and to what extent, MEM (https://biit.cs.ut.ee/mem/index.cgi) was adopted, where the human A-AFFY-44 dataset was selected for coexpression analysis of genes.

### 2.13. Statistical Analyses

All statistical analysis was completed using GraphPad Prism 8.0 (USA). Data were described by means ± standard deviations (SD). Normally distributed data were determined by one-way analysis of variance (ANOVA), followed by the Tukey post hoc test. Not normally distributed data were analyzed with nonparametric Wilcoxon tests. Survival data were analyzed using the Kaplan–Meier method, and the survival curves were compared using the log-rank test. Statistical significance was defined as *P* value < 0.05.

## 3. Results

### 3.1. PFKFB3 Is Upregulated in the Small Intestines of CLP Mice

**Figure 1:**
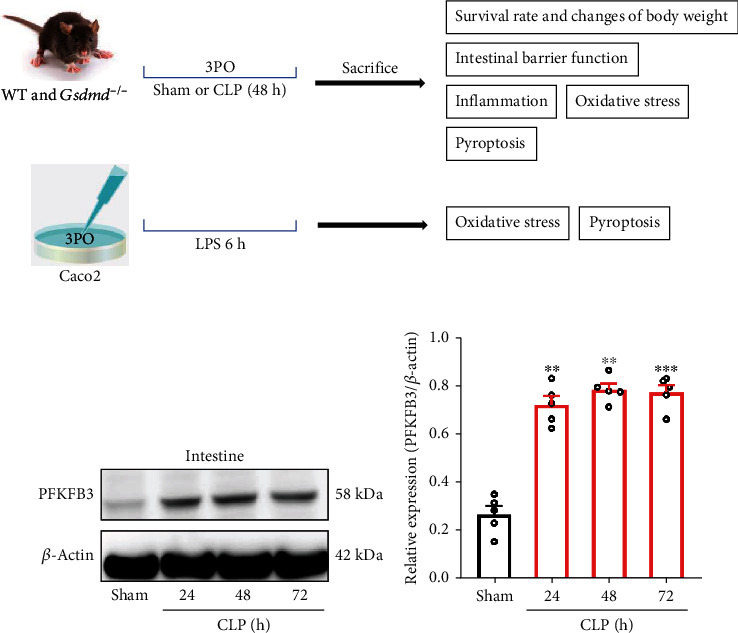
The expression of PFKFB3 was elevated in the intestinal tissues of septic mice. (a) The experimental design of this study. (b, c) The expression of PFKFB3 was analyzed by Western blot at different time points after CLP. Experimental values are expressed as means ± SD (*n* = 5 per group). Statistical analysis was performed using one-way ANOVA (c). ^∗∗^*P* < 0.01 and ^∗∗∗^*P* < 0.001.

PFKFB3 expression has been reported to be elevated in the septic myocardium. Further, inhibition of PFKFP3 alleviates myocardial injury and acute lung injury in septic rat models [7; 13]. Therefore, we hypothesized that PFKFB3 was upregulated in septic intestinal tissues. To test this hypothesis, we developed a mouse model of sepsis (Figure 1(a)). Western blot analysis showed that the expression of PFKFB3 was significantly increased in the intestinal tissue of mice at 24, 48, and 72 hours after CLP operation (Figures 1(b) and 1(c)). Thus, we hypothesized that PFKFB3 might contribute significantly to CLP-induced intestinal injury.

### 3.2. Inhibition of PFKFB3 Improves Survival in CLP Mice

**Figure 2:**
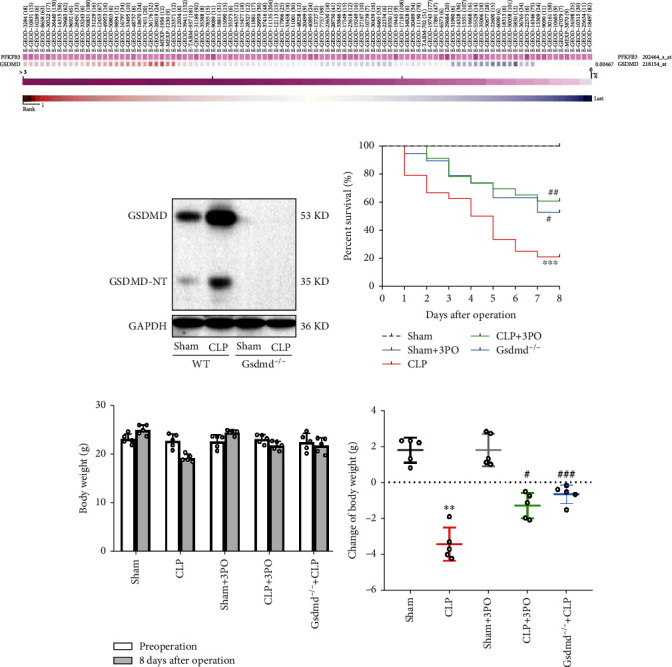
Inhibition of PFKFB3 and Gsdmd gene knockout can improve the prognosis of septic mice. (a) The coexpression of PFKFB3 and GSDMD was analyzed by MEM. (b, c) The LPS-induced PFKFB3 expression was inhibited by 3PO in Caco-2 cells. (d) GSDMD and GSDMD-NT expression in intestinal tissues of wild-type and Gsdmd^−/−^ mice after sham or CLP operation. (e) Survival rates were calculated in different groups. The values were shown as mean ± SD (*n* = 5 per group in (d) and (e)). Statistical analysis was performed using a *t*-test (e) and log-rank test (c). ^∗∗∗^*P* < 0.001 versus sham group; ^#^*P* < 0.05, ^##^*P* < 0.01, and ^###^*P* < 0.001 versus CLP group.

MEM analysis showed that PFKFB3 and GSDMD were coexpressed in some cases (Figure 2(a)). Deletion of the Gsdmd gene has been reported to alleviate sepsis-induced intestinal barrier dysfunction [17]. Therefore, we evaluated the effect of 3PO and Gsdmd gene deletion on survival in CLP-operated mice. The mice were divided into five groups and monitored for eight days. The survival rate in the CLP group (5 out of 24 mice, 20.8%) was significantly lower than that of the sham-operated group and sham+3PO group (10, 100% sham-operated group and sham+3PO group, *P* < 0.001). Interestingly, the CLP+3PO and CLP+Gsdmd^−/−^ groups had significantly increased survival rates at 60.9% (14 out of 23 mice, *P* < 0.01) and 52.7% (10 out of 19 mice, *P* < 0.05), respectively (Figure 2(c)). In addition, a comparative analysis of the body weight in the surviving mice showed that 3PO and GSDMD gene deletion significantly increased the body weight of the septic mice (*P* < 0.05 and *P* < 0.001, respectively) (Figures 2(d) and 2(e)).

### 3.3. The Effect of 3PO on the Intestinal Pathological Changes and Permeability

**Figure 3:**
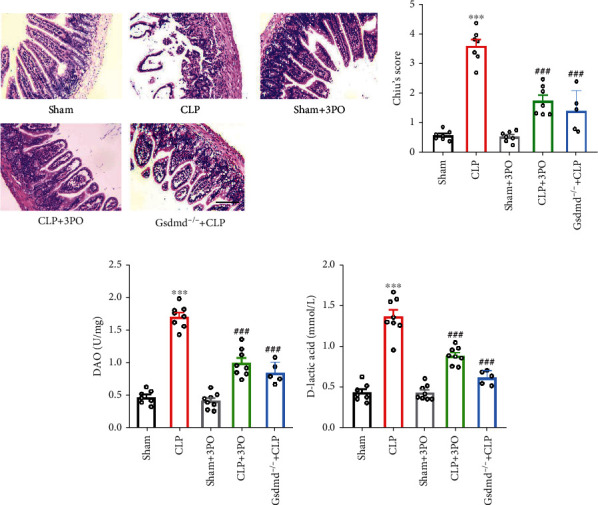
Effects of 3PO or Gsdmd genetic deletion on intestinal histology and intestinal barrier function. At 24 h after sham or CLP operation, the histopathological alterations of gut tissues were measured using H-E staining (a) and analyzed by Chiu's score (b). Scale bar = 100 *μ*m. (c) Detection of DAO and (d) D-lactic acid levels in serum. The values were shown as mean ± SD (*n* = 7 per group). Statistical analysis was performed using a *t*-test (b–d). ^∗∗∗^*P* < 0.001 versus sham group; ^###^*P* < 0.001 versus CLP group.

Light microscopy showed that the villi in the small intestinal mucosa of the sepsis mice were thinner, shorter, and more disorderly arranged, with cell shedding at the top of the villi, consistent with previous studies [17, 18]. The microvilli became thinner and shorter, and some microvilli were destroyed (Figure 3(a)). The results also showed that 3PO and Gsdmd gene deletion significantly improved the pathologic features of intestinal injury and decreased Chiu's score (*P* < 0.001) (Figure 3(b)). These results suggested that 3PO and Gsdmd gene deletion could significantly reduce the pathological manifestations of CLP-induced intestinal injury.

Sepsis can lead to increased intestinal permeability in mice, an important cause of bacteremia and septic shock [2, 4]. D-lactic acid and DAO are important indicators for intestinal damage, with reductions in D-lactic acid and DAO indicating an impairment in the integrity of the intestinal mucosa [19, 20]. The results of this study showed the serum levels of D-lactic acid and DAO to be markedly increased in mice after CLP operation. On the other hand, 3PO and Gsdmd gene deletion significantly reduced D-lactic acid and DAO expression, suggesting that 3PO and Gsdmd gene deletion could significantly improve the intestinal permeability induced by CLP (*P* < 0.001) (Figures 3(c) and 3(d)).

### 3.4. The Effect of 3PO on Intestinal Inflammatory Cytokines

**Figure 4:**
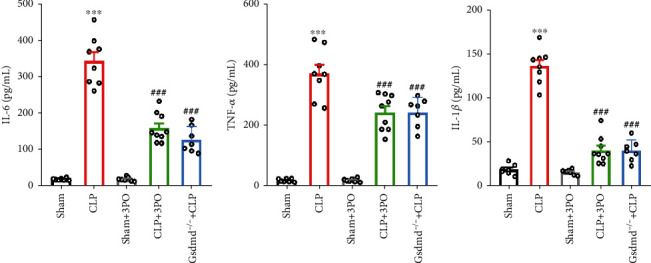
Effect of 3PO and Gsdmd gene knockout on inflammatory cytokines in intestinal tissue. (a–c) Levels of IL-6, TNF-*α*, and IL-1*β* in small intestinal tissues were measured by ELISA. The values were shown as mean ± SD (*n* = 6 − 9). ^∗∗∗^*P* < 0.001 versus sham group; ^###^*P* < 0.001 versus CLP group. The values were shown as mean ± SD (*n* = 5). Statistical analysis was performed using a *t*-test (a–c). ^∗∗∗^*P* < 0.001 compared with the no-treatment group and ^##^*P* < 0.01 compared with the CLP treatment group.

Elevated inflammatory mediators in the intestinal tract are an important cause of intestinal injury [4, 17]. According to recent studies, 3PO is suggested to protect against acute lung injury by reducing inflammation. Therefore, ELISA was used to detect the levels of TNF-*α*, IL-6, and IL-1*β* in each group. The results showed that TNF-*α*, IL-6, and IL-1*β* levels in the intestine of the CLP group were significantly increased compared with the sham-operated group (*P* < 0.05). However, 3PO and Gsdmd gene deletion significantly reduced the levels of TNF-*α*, IL-6, and IL-1*β* in intestinal tissues of septic mice (*P* < 0.05) (Figures 4(a)–4(c)).

### 3.5. 3PO Inhibits the Expression of NLRP3 in Septic Intestinal Tissue

**Figure 5:**
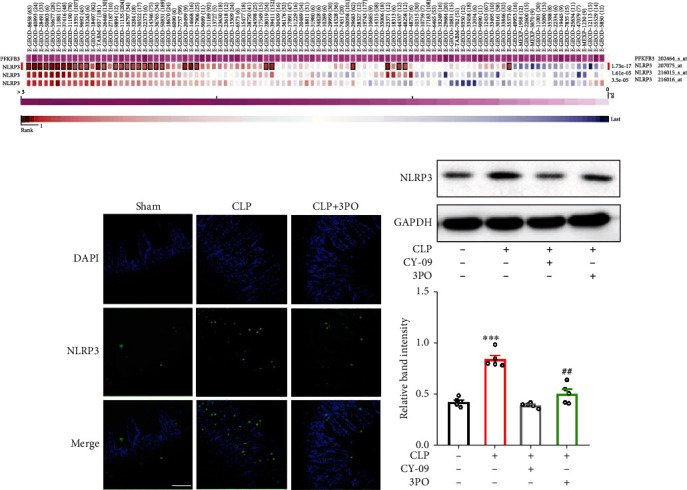
3PO downregulates NLRP3 expression. The coexpression of PFKFB3 and NLRP3 was analyzed by MEM (a). The expression of NLRP3 was detected in small intestinal tissues by immunofluorescence (b) and Western blot (c). The values were shown as mean ± SD (*n* = 5). Statistical analysis was performed using a *t*-test (c). ^∗∗∗^*P* < 0.001 compared with the no-treatment group and ^##^*P* < 0.01 compared with the CLP treatment group.

Based on the above results, we speculated that 3PO could reduce intestinal barrier dysfunction by inhibiting GSDMD-dependent pyroptosis. Then, we further explored the effect of 3PO on cell pyroptosis. Activation of NLRP3 inflammasome is an essential pathway of cell pyroptosis [21]. MEM analysis showed that PFKFB3 and GSDMD were coexpressed in some cases (Figure 5(a)). Therefore, we explored the effect of 3PO on NLRP3 expression. Both fluorescence staining and Western blot indicated that the expression of NLRP3 was significantly increased in the intestinal tissues of septic mice. Furthermore, 3PO could significantly inhibit the expression of NLRP3 (Figures 5(b) and 5(c)). These results suggest that 3PO may alleviate intestinal barrier dysfunction by inhibiting NLRP3-dependent cell pyroptosis.

### 3.6. 3PO Protected against Sepsis-Induced Intestinal Injury and Pyroptosis

**Figure 6:**
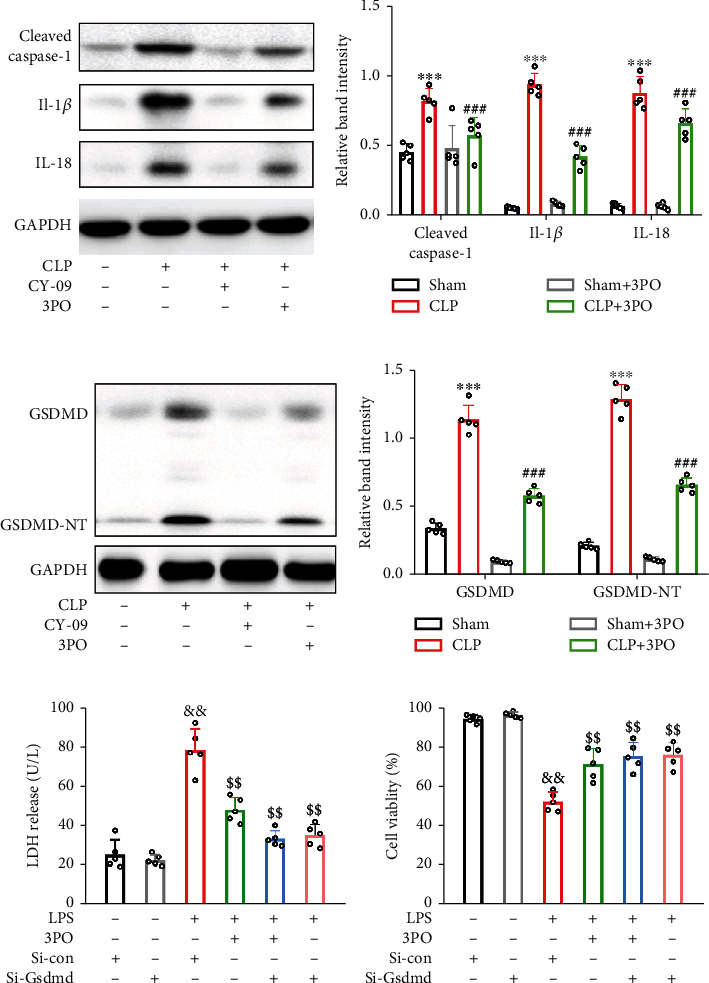
3PO protects against pyroptosis in vitro and in vivo. (a, b) Pyroptosis-related proteins, including NLRP3, cleaved caspase-1, IL-1*β*, and IL-18, were examined by Western blot in intestinal tissue of mice. (c, d) The expression of GSDMD was detected by Western blot in intestinal tissue of mice. si-Gsdmd was used to inhibit pyroptosis and reveal the relationship between 3PO and pyroptosis. (e) Levels of the released LDH were detected in C2C12 culture medium supernatant. (f) Cell viability was determined using the CCK-8 assay. The values were shown as mean ± SD (*n* = 5). Statistical analysis was performed using a *t*-test (b, d–f). ^∗∗∗^*P* < 0.001 compared with the no-treatment group and ^###^*P* < 0.001 compared with the CLP treatment group. ^&&^*P* < 0.01 compared with the si-Con group and ^$$^*P* < 0.01 compared with the LPS+si-Con group. 3PO protected against CLP-induced intestinal injury by suppressing NLRP3/caspase-1/GSDMD.

To determine whether 3PO played a protective role by inhibiting NLRP3-induced pyroptosis, the CLP mice were given 3PO in the model group and CY-09, an NLRP3 inhibitor, as the positive control. IL-1*β* and IL-18 are the typical inflammatory cytokines released from the pyroptotic cell. Western blot analysis showed that treatment with 3PO and CY-09 significantly reduced cleaved caspase-1 and pyroptosis-associated inflammatory mediators, IL-1*β* and IL-18 (Figures 6(a) and 6(b)). In addition, GSDMD (full-length GSDMD) and its cleaved and activated fragment GSDMD-NT were significantly decreased in the 3PO and CY-09 groups (Figures 6(c) and 6(d)). 3PO has been shown to regulate many forms of cell death. We further inhibited pyroptosis by knocking down Gsdmd in Caco-2 and established the sepsis cell model with LPS to confirm the role of 3PO in cell pyroptosis in intestinal tissues of CLP. The results revealed that in the absence of 3PO, a knockdown of Gsdmd also improved LDH release and cell viability (Figures 6(e) and 6(f)). Caco-2 cells were transfected with si-GSDMD to knock down the expression of GSDMD (Supplementary Figure 1). There were no differences in cell viability and LDH between the si-Gsdmd +3PO and si-Gsdmd groups (Figures 6(e) and 6(f)). In conclusion, treatment with 3PO could inhibit the NLRP3-induced pyroptosis, one of the primary cell death pathways in intestinal injury in sepsis.

### 3.7. Effect of 3PO on Oxidative Stress in Intestinal Tissues

**Figure 7:**
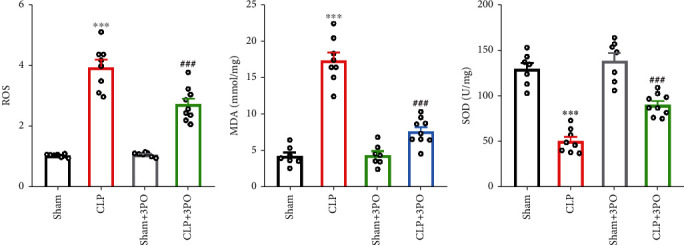
3PO alleviates oxidative stress in intestinal tissues. (a–c) Oxidative stress was measured by ROS, MDA, and SOD. The values were shown as mean ± SD (*n* = 7 − 9). Statistical analysis was performed using a *t*-test (a–c). ^∗∗∗^*P* < 0.001 compared with the sham group and ^###^*P* < 0.001 compared with the CLP treatment group.

Aerobic glycolysis has been reported to be associated with increased oxidative stress in cancer [22]. Therefore, we investigated whether 3PO affects oxidative stress in intestinal tissue. The results showed that the ROS and MDA levels were decreased, while SOD levels were increased significantly after treatment with 3PO (Figures 7(a)–7(c)).

### 3.8. 3PO Inhibits NLRP3/Caspase-1 Pyroptosis by Suppressing ROS

**Figure 8:**
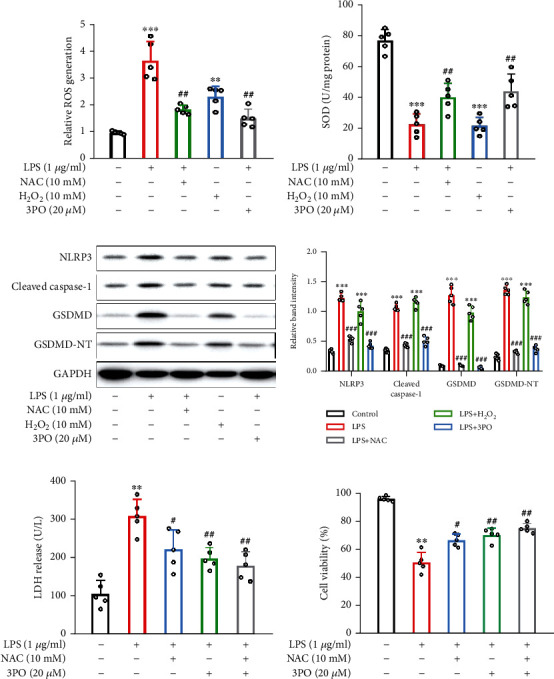
3PO suppressed pyroptosis by inhibiting ROS. (a, b) Oxidative stress was measured by ROS and SOD in vitro. (c, d) Pyroptosis-related proteins were detected by Western blot. (e) The released levels of LDH were detected. (f) Cell viability was measured using the CCK-8 assay. The values were shown as mean ± SD (*n* = 5). Statistical analysis was performed using a *t*-test (a, b, d–f). ^∗∗∗^*P* < 0.001 compared with the no-treatment group and ^###^*P* < 0.001 compared with the LPS treatment group.

Oxidative stress is another important cause of intestinal damage and intestinal barrier dysfunction in sepsis. Numerous reports have suggested that ROS is an important activator of NLRP3 [14, 23]. Therefore, we stimulated Caco-2 cells with LPS and intervened with NAC (an ROS scavenging agent) and 3PO. Results showed that LPS induced the upregulation of ROS and LDH and decreased SOD and cell viability. However, 3PO can significantly reverse this change, and the effect is similar to NAC (Figures 8(a), 8(b), 8(e), and 8(f)). In addition, both LPS and H_2_O_2_ induced activation of the NLRP3/caspase-1/GSDMD pathway in Caco-2 cells, apparently reversed by NAC and 3PO (Figures 8(c) and 8(d)). These results demonstrate that 3PO protects against sepsis-induced intestinal barrier dysfunction and damage by inhibiting the ROS-NLRP3 pathway.

## 4. Discussion

Sepsis is a leading cause of death in noncardiac intensive care units worldwide. It is characterized by rapid disease progression and high mortality [1]. Intestinal mucosal dysfunction is considered a driving factor for multiple organ dysfunction syndromes [2, 4]. Therefore, there is an urgent need to explore new and potential therapeutic targets to ameliorate or prevent the progression of this devastating disease. A growing number of studies have shown that PFKFB3 is enhanced in various inflammatory or immune-related diseases, such as acute lung injury, myocardial dysfunction, and cancer [7, 10, 13, 22]. In this study, the Western blot results showed that PFKFB3 was significantly elevated in septic mice with intestinal injury (Figure 1). To further investigate the role of PFKFB3 in sepsis-induced intestinal dysfunction, we established CLP mice and intervened with the PFKFB3 inhibitor 3PO. 3PO was shown to inhibit inflammation, oxidative stress, and pyroptosis, thereby improving intestinal dysfunction and alleviating sepsis.

Inflammation has long been recognized as an important pathological mechanism leading to organ injury in sepsis. Previous studies have shown a complex relationship between intestinal mucosal injury and immune homeostasis [24, 25]. PFKFB3 plays an important role in regulating inflammation and cell death in a variety of inflammatory disease models [7, 12, 13]. Recently, it was reported that the pyroptosis pathway contributed to intestinal injury induced by bacterial sepsis [17]. MEM analysis showed that PFKFB3 and GSDMD were significantly coexpressed. Therefore, we explored the effects of Gsdmd gene deletion and PFKFB3 inhibitor, 3PO, on intestinal damage in sepsis. First, we found that Gsdmd gene deletion and 3PO could significantly improve the nutritional status and prognosis of sepsis mice. The HE staining showed that 3PO and Gsdmd gene deletion could significantly improve the intestinal mucosal injury caused by CLP. Further, we explored the effect of 3PO on the levels of inflammatory mediators in the small intestinal tissues. We found that TNF-*α*, IL-6, and IL-1*β* were significantly increased in the sepsis group, offset by 3PO and Gsdmd gene deletion. These results were consistent with previous reports [7, 26]. These results indicate that 3PO could significantly reduce intestinal inflammation.

Intestinal flora D-lactic acid and mammalian intestinal mucosa high enzyme DAO are sensitive markers of intestinal mucosal injury and barrier function. In this study, serum levels of D-lactic acid and DAO in the sepsis group were significantly higher than in the sham operation group, indicating an impaired intestinal mucosal barrier function in sepsis rats. The serum levels of D-lactic acid and DAO in mice treated with 3PO and Gsdmd gene deletion were significantly decreased, suggesting that 3PO and Gsdmd gene deletion could enhance the intestinal barrier function. In sepsis, damage to the intestinal mucosa and barrier dysfunction leads to bacteria and toxins passing into the bloodstream, exacerbating the condition. This study revealed that 3PO had a substantial protective effect on the intestinal mucosal barrier.

Numerous studies have shown that cell pyroptosis, a programmed cell death dependent on GSDMD activation, is closely related to the systemic inflammatory response and intestinal barrier dysfunction caused by sepsis [17, 27, 28, 29]. Activation of the NLRP3 inflammasome leads to pro-caspase-1 self-cleavage and the production of active c-caspase-1, which in turn mediates the maturation and secretion of IL-1*β* and IL-18. In addition, activated c-caspase-1 cleaved GSDMD to produce GSDMD-NT with pore-forming activity, leading to cell lysis and inflammatory cytokine release [28, 29, 30, 31]. During bacterial infection, GSDMD is necessary for cell pyroptosis, acts as a pathway for the release of danger signals such as IL-1 family cytokines, and eliminates the replicative niche of bacterial [27]. However, overactivity of GSDMD can aggravate inflammation, leading to septic shock [28, 32, 33, 34]. Several studies have found that GSDMD is an essential therapeutic target for reducing the inflammatory response and organ dysfunction in sepsis [26, 27, 34, 35, 36, 37]. GSDMD is highly expressed in intestinal epithelial cells, while caspase-1 and caspase-11 play an important role in controlling intestinal pathogens [38, 39]. Furthermore, GSDMD contributes to NLRP9-dependent pyroptosis in intestinal epithelial cells (IECs) in mice infected with rotavirus [40]. A recent study reported that the pyroptotic pathway contributed to inflammatory intestinal injury induced by bacterial sepsis [17]. A recent study has found that inhibition of the trophoblast TLR4/NF-*κ*B/PFKFB3 signaling pathway to correct glycometabolic reprogramming and NLRP3 inflammation-induced pyroptosis may be a treatment approach for preeclampsia [41]. In intestinal I/R injury models, metformin inhibits TXNIP expression and TXNIP interaction with NLRP3, protecting intestinal barrier destruction and pyroptosis [42]. However, whether 3PO can inhibit pyroptosis in sepsis remains unclear. In this study, 3PO was shown to significantly inhibit the overexpression of NLRP3/caspase-1/GSDMD in intestinal epithelial cells induced by CLP. These results suggest that 3PO protects against sepsis-induced intestinal injury by suppressing NLRP3/caspase-1/GSDMD-dependent pyroptosis.

Oxidative stress plays a critical role in regulating various biological processes, including cell death and immunity [43, 44]. Oxidative stress has also been reported to be an essential initiator of pyroptosis and is closely related to septic organ failure [23, 45, 46]. Oxidative stress is also a key factor in intestinal barrier dysfunction in patients with sepsis [47]. ROS and MDA are often used as biomarkers for oxidative damage in diseases. SOD is one of the most important biological enzymes in the antioxidant system. This study found that ROS and MDA were significantly increased while SOD activity was low in the sepsis group. However, 3PO partially reversed this phenomenon. This finding suggests a protective effect of PFKFB3 inhibitor against intestinal oxidative stress.

**Figure 9:**
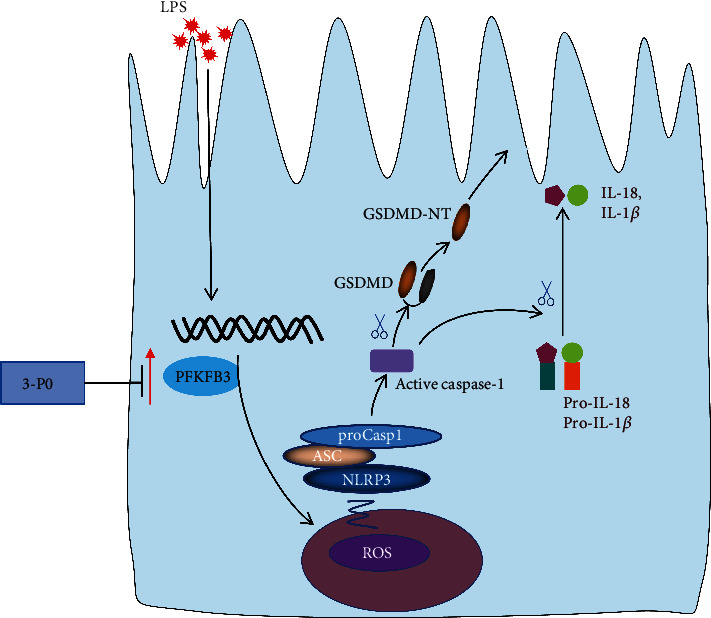
Schematic model for the inhibition of PFKFB3 preserves intestinal barrier function in sepsis via preventing GSDMD-dependent pyroptosis by inhibiting ROS production.

ROS are a group of molecules that include peroxides, superoxides, hydroxyl groups, and singlet oxygen [48]. As a second messenger driving inflammasome activation, ROS is considered a common NLRP3/caspase-1 complex activator that mediates pyroptosis [48]. Under normal circumstances, antioxidant enzymes can clear ROS during cell metabolism, maintaining a balance between ROS production and clearance. ROS accumulation may overwhelm the endogenous antioxidant defense system and thus cell disorders such as oxidative stress upregulation and cell death [48]. Studies have shown that LPS can induce intestinal epithelial cell damage through ROS production [45, 47, 49]. A growing number of studies have found that ROS interacts with NLRP3 inflammasome during sepsis and regulates immune-inflammatory response [23, 44, 48]. A recent study found that targeting PFKFB3 to block glycolysis alleviates acute lung injury associated with sepsis by inhibiting inflammation, ROS production, and apoptosis of alveolar epithelial cells [7]. In this study, we found that LPS-stimulated NLRP3 inflammasomes significantly increased in a ROS-dependent manner, inducing cell death. We also found that 3PO and NAC inhibited ROS production, thereby reducing NLRP3/caspase-1/GSDMD activation and thus alleviating cell damage. However, H_2_O_2_ reversed the protective effect of 3PO on pyroptosis. These results indicate that 3PO mainly inhibited cell death by inhibiting ROS production and thus played a protective role in the intestinal tract (Figure 9).

## 5. Conclusion

In conclusion, this study suggests that Gsdmd gene knockout has a protective effect on intestinal barrier function in sepsis. The mechanism of this effect may be related to the inhibition of inflammation and oxidative damage. The findings of this study provide insights into the role of GSDMD in regulating intestinal barrier function in sepsis and provide a potential therapeutic target for the treatment of sepsis.

## Data Availability

The raw data supporting the conclusions of this article will be made available by the authors, without undue reservation.

## Supplementary Materials

Supplementary Materials Supplementary Figure 1: siRNA targeting GSDMD reduced GSDMD-N protein expression in Caco-2 cells. (A) Western blot was used to measure the expression of GSDMD-N protein. (B) Quantitative analysis of GSDMD-N protein was performed. The values were shown as mean ± SD (*n* = 5). ^∗∗^*P* < 0.01 compared with the control group and ^##^*P* < 0.01 compared with the LPS si-con treatment group.
