# Supplementary material for: Inhibition of PFKFB Preserves Intestinal Barrier Function in Sepsis by Inhibiting NLRP3/GSDMD
Source: Oxid Med Cell Longev. 2022 Dec 23;2022:8704016. doi: 10.1155/2022/8704016 (PMC9803577; doi:10.1155/2022/8704016)

**Supplementary Figure 1** siRNA targeting GSDMD reduced GSDMD-N protein expression in Caco-2 cells. A: Western blot was used to measure the expression of GSDMD-N protein. B: Quantitative analysis of GSDMD-N protein was performed. The values were showed as the mean ± SD (n = 5). **p < 0.01 compared with control group and ##p < 0.01 compared with LPS si-con treatment group.


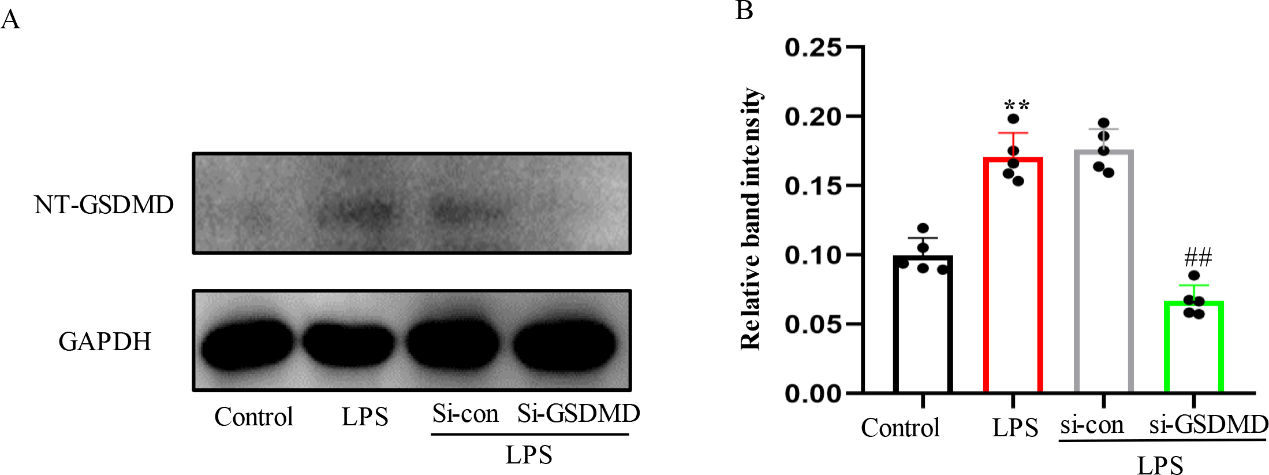

Supplement: Supplementary Materials — Supplementary Figure 1: siRNA targeting GSDMD reduced GSDMD-N protein expression in Caco-2 cells. (A) Western blot was used to measure the expression of GSDMD-N protein. (B) Quantitative analysis of GSDMD-N protein was performed. The values were shown as mean ± SD (n = 5). ∗∗P < 0.01 compared with the control group and ##P < 0.01 compared with the LPS si-con treatment group. [file 8704016.f1.docx]
